# Community Diversity of Fungi Carried by Four Common Woodpeckers in Heilongjiang Province, China

**DOI:** 10.3390/jof10060389

**Published:** 2024-05-29

**Authors:** Wenhui Shi, Iram Maqsood, Keying Liu, Meichen Yu, Yuhui Si, Ke Rong

**Affiliations:** 1 College of Wildlife and Protected Area, Northeast Forestry University, Harbin 150040, China; 2 Department of Zoology, Shaheed Benazir Bhutto Women University Peshawar Pakistan, Peshawar 25000, Pakistan; 3 Key Laboratory of Wildlife Conservation Biology, National Forestry and Grassland Administration, Beijing 100013, China

**Keywords:** α diversity, β diversity, high-throughput sequencing, ITS rRNA, fungi, contour feathers

## Abstract

Woodpeckers exhibit selectivity when choosing tree cavities for nest development in forest ecosystems, and fungi play a significant and important role in this ecological process. Therefore, there is a complex and intricate relationship between the various behaviors of woodpeckers and the occurrence of fungal species. Research into the complex bond between fungi and woodpeckers was undertaken to provide more information about this remarkable ecological relationship. Through the process of line transect sampling, woodpecker traces were searched for, and mist nets were set up to capture them. A total of 21 woodpeckers belonging to four species were captured. High-throughput sequencing of the ITS region was performed on fungal-conserved samples to enable an in-depth analysis of the fungal communities linked to the woodpeckers’ nests. Members of Ascomycota were the most abundant in the samples, accounting for 91.96% of the total, demonstrating the importance of this group in the forest ecosystem of this station. The statistical results indicate significant differences in the fungal diversity carried by woodpeckers among the different groups. Species of *Cladosporium* were found to be the most prevalent of all the detected fungal genera, accounting for 49.3%. The top 15 most abundant genera were *Cladosporium*, *Trichoderma*, *Beauveria*, *Epicococcum*, *Hypoxylon*, *Penicillium*, *Nigrospora*, *Aspergillus*, *Oidiodendron*, *Cercospora*, *Talaromyces*, *Phialemo-nium*, *Petriella*, *Cordyceps*, and *Sistotrema*. The standard Bray–Curtis statistical technique was used in a hierarchical clustering analysis to compute inter-sample distances, allowing for the identification of patterns and correlations within the dataset. We discovered that in the grouped samples from woodpeckers, there were differences in the diversity of fungal communities carried by four woodpecker species, but the less dominant fungal species were still similar. The findings highlight the need to consider these diverse ecological linkages in woodpecker research and conservation efforts.

## 1. Introduction

In ecology, amidst growing concerns such as climate change and the introduction of non-native species, understanding the mechanisms facilitating species coexistence remains paramount [1]. With biodiversity conservation gaining traction, the cohabitation of different species has emerged as a central inquiry worldwide [2,3]. Various studies have explored the multifaceted relationship between microorganisms and animals, encompassing influences on host evolution, metabolic rates, and yet-to-be-discovered interactions [4,5]. Different aspects of the relationship between microorganisms and animals have been expressed in various ways, such as the influence on host evolution of animals [6,7] and the disruption of host metabolic rates [7], but there are some that are still unknown.

China is one of the countries with the greatest bird resources in the world, accounting for roughly 14 percent of the global bird population [8]. Northeast China’s geographical location holds significant importance in terms of avian habitats and reproductive processes [9]. The observed changes in avian ecological variety and intricate migratory patterns serve as compelling indications of the significant role played by Northeast China in the bird ecosystem.

Birds, owing to their extensive migratory journeys, serve as carriers for zoonotic infections, impacting both their distribution and the phylogeography of pathogens. Beyond transmitting diseases, birds play a crucial role in dispersing fungal spores through activities like feeding and pecking [1,10,11]. Also, regarding the association between woodpeckers and fungi and not birds and zoonotic infections, among avian species, woodpeckers stand out as significant agents of fungal spore dissemination and colonization [12,13]. Furthermore, the ecological roles of fungi have a substantial influence on bird behaviors, particularly those of different woodpecker species, affecting their feeding habits and nest construction behaviors. As indicator and umbrella species, woodpeckers contribute to assessing forest ecosystem health and conserving these habitats by controlling forest pests and providing nesting sites for numerous avian and mammalian species [14,15,16]. Their significance lies in their potential role as bio-controllers of forest pests, as they prey on these pests [17,18]. Furthermore, they provide habitats for many avian and mammalian species, allowing them to establish roosting and breeding grounds [19]. Consequently, the examination of the ecological features associated with woodpeckers plays a significant role in understanding the patterns of forest ecosystem transitions and conserving these ecosystems [20].

Wood fungi, known for inducing softness and decay in trees, play a pivotal role in woodpecker habitat selection by rendering a site, branch, or trunk soft and malleable [21,22,23]. As a result, woodpeckers exhibit selectivity in their choice of trees for nesting within their forest habitats, with fungi playing a distinct and significant part in this ecological process. Red-cockaded woodpeckers (*Picoides borealis*) in the Holarctic region have a predilection for selecting tree trunks that have been colonized by rotting fungus as nesting sites, as shown by Jusino et al. [24]. Nevertheless, a recent study conducted by Jusino et al. posits the existence of a mutually advantageous and symbiotic association between woodpeckers and fungi. According to Jusino et al. [19], woodpeckers engage in the process of tree excavation subsequent to the softening of trees by fungus. Additionally, woodpeckers play a crucial role in facilitating successful communication for the fungi. 

The nesting preferences of woodpeckers often involve living trees, snags, or rotting branches, and the practice of establishing new nests annually is often observed [25,26]. The construction of a new nest by the majority of woodpecker species typically requires a duration of two to six weeks [27,28,29]. 

In contrast to the outer environment, nests offer a relatively constant micro-environment and mitigate fluctuations in interior temperature for the organisms inhabiting them [30,31]. The relative humidity within the cave exhibits a significant increase of over 90% compared to the external environment, and this elevated level remains relatively constant throughout the length of the day, indicating the absence of substantial variations in numerical values [32,33].

In contrast to other avian species that construct primary and secondary cavities, woodpeckers exhibit a distinct nesting behavior wherein they eschew the use of additional materials as nesting substrates, instead opting to deposit their eggs directly within the base of a burrow. According to Jackson et al. [34], the presence of a limited quantity of wood debris at the base of the nest hole suggests that the softened wood debris also contributes to the brooding process within the nest hole. Multiple studies have indicated that the nesting behavior of woodpeckers, characterized by their brooding in nest holes, facilitates the colonization and flourishing of various fungi [35,36,37]. 

In this study, four species of woodpecker, the lesser spotted woodpecker (*Dendrocopos minor*), the great spotted woodpecker (*Dendrocopos major*), the white-backed woodpecker (*Dendrocopos leucotos*), and the grey-headed green woodpecker (*Picus canus*), were analyzed. These species are members of the woodpecker family that carry out the primary function of nest cavity excavation. Furthermore, mist nets have been employed for several decades in the field of ornithological research [38]. In avian studies, investigations pertaining to autecology and community dynamics sometimes necessitate the capture of individual birds. Consequently, mist nets have emerged as commonly employed tools for achieving this specific objective, constituting the primary instrument employed in surveys focused on the capturing of terrestrial birds.

Bird species surveys often rely on the use of mist nets as a valuable complement to visual and auditory methods [39]. Mist nets are widely recognized as important supplementary sampling tools in studies focused on bird communities [40] and inventories [41]. The utilization of mist nets for individually marking birds is favored by researchers due to its ability to facilitate studies that involve the estimation of home ranges, population levels, and patterns [42]. Furthermore, the data obtained from mist net captures exhibit obvious biases since there are variations in the success rates of capturing different bird species [43].

Recent studies suggest that there is a mutually beneficial symbiotic association between woodpeckers and fungi, wherein woodpeckers excavate trees softened by fungi, facilitating both nesting and fungal dissemination. Similarly, the investigation of the association between woodpeckers and their nests primarily relies on the observation of visual cues [22,23,44,45]. This experiment was carried out on Mao’er Mountain in the old mountain area in Harbin, Heilongjiang Province, where contour feathers from woodpeckers were collected. In this study, we utilized high-throughput molecular sequencing technology as a means of investigating the fungal species harbored by woodpeckers. The objectives of this study were to (i) utilize high-throughput molecular sequencing technology to identify fungal species harbored by woodpeckers in the Mao’er Mountain region, (ii) analyze the diversity and abundance of fungal species associated with Palearctic woodpeckers, and (iii) assess the relationship between woodpecker species and the presence of specific fungal taxa to contribute a better understanding of the ecological dynamics between woodpeckers and fungi in forest ecosystems.

## 2. Materials and Methods

### 2.1. Field Sampling

Field sampling was carried out on Mao’er Mountain in the old mountain area in Harbin, Heilongjiang Province (45°20′–45°25′ N, 127°30′–127°34′ E), during the ecological season, specifically from 10 March to 25 May and from 5 September to 20 November, in 2021. When traces of woodpeckers or the sounds of their pecking were detected during the transect survey, immediate and precise location detection was carried out using binoculars, and information such as species, gender, and activity height was recorded. Once the active area of a woodpecker was pinpointed, suitable locations for setting up mist nets were chosen, typically being positioned beneath or around trees in which woodpeckers were frequently active. Upon capturing a woodpecker, marking and sampling procedures were conducted. As a standard procedure, the captured avian specimens were carefully recorded, and their information was consistently documented and stored on a computer.

In this experiment, woodpeckers were designated as the focal avian species. The protocol for this investigation was developed based on the methodology outlined by V. Javůrková in her previous experiments [46]. Upon capturing a woodpecker in a net, the researchers promptly donned sterile disposable gloves that were carefully chosen to match the dimensions of their hands. This measure facilitated the safe and efficient removal of the woodpecker from the net, minimizing the risk of contamination. The individuals assigned to the task of handling the woodpeckers and other avian species in the mist nets had undergone comprehensive training and have considerable expertise in the safe management of birds. Following the removal of each woodpecker, a careful procedure was undertaken to remove 1–2 contour feathers. These feathers were gently plucked using tweezers and subsequently transferred into a previously sterilized centrifuge tube. Feathers obtained from a single avian specimen were merged into a single container and did not need to be separated. Prior to the experiment, both the centrifuge tubes and tweezers were subjected to high-temperature sterilization. Following the completion of the sampling process, all collected samples were kept at a temperature of 4 °C and subsequently transferred to the laboratory within a time frame of 24 h for further analysis using high-throughput sequencing techniques. Following the sampling of the woodpeckers, data collection continued in accordance with established ringing protocols, and the corresponding data were securely recorded.

### 2.2. DNA Extraction and PCR Amplification

Microbial DNA was extracted using the E.Z.N.A.^®^ DNA Kit (Omega Bio-tek, Norcross, GA, USA). The extraction process was performed strictly in accordance with the protocols provided by the manufacturer. Once the extracted amplicons had been obtained and purified, they were subjected to further analysis. The DNA concentration of each purified amplicon was measured using a Qubit^®^ fluorometer (Thermo Fisher Scientific, Waltham, MA, USA) and normalized to the same concentration. Subsequently, equal amounts of each amplicon were pooled to create a composite library for sequencing.

PCR was performed to amplify the V4-V5 region of the fungal ribosomal RNA gene. The PCR cycling conditions involved an initial denaturation at 95 °C for 2 min, followed by 25 cycles of denaturation at 95 °C for 30 s, annealing at 55 °C for 30 s, extension at 72 °C for 30 s, and a final extension at 72 °C for 5 min. The primers used in the PCR reaction were 515F (5′-barcode-GTGCCAGCMGCCGCGG-3′) and 907R (5′-CCGTCAATTCMTTTRAGTTT-3′). Here, each barcode represents a unique eight-base sequence assigned to each sample. Three replicate PCR reactions were performed, with each reaction containing a 20 μL mixture consisting of 4 μL of 5× FastPfu Buffer, 2 μL of 2.5 mM dNTPs, 0.8 μL of each primer (5 μM), 0.4 μL of FastPfu Polymerase, and 10 ng of template DNA. Following amplification, the resulting amplicons were extracted from 2% agarose gels and purified using the AxyPrep DNA Gel Extraction Kit (Axygen Biosciences, Union City, CA, USA), following the instructions provided by the manufacturer (Shanghai Biozeron Biotech. Co., Ltd., Shanghai, China).

The composite library was then paired-end sequenced using the Illumina MiSeq platform (Illumina Inc., San Diego, CA, USA) and the MiSeq Reagent Kit v3 (600 cycles) according to the manufacturer’s instructions. The generated sequencing reads were subjected to quality control, which included filtering out low-quality reads, removing adapter sequences, and eliminating reads containing ambiguous nucleotides.

Bioinformatics analysis was performed on the high-quality sequencing data using the Quantitative Insights into Microbial Ecology 2 (QIIME2) software package, we utilized the software accessed from https://qiime2.org/ and accessed on 1 February 2022. Operational taxonomic units (OTUs) were selected based on a 97% sequence similarity threshold using the DADA2 algorithm. Taxonomic classification of the OTUs was accomplished using the Silva database (version 138) as a reference.

The resulting taxonomic data were further analyzed to determine the alpha and beta diversity of the microbial communities. Alpha diversity measures, such as the Shannon diversity index and values from observed species analysis, were calculated to assess the microbial richness and evenness within each sample, while beta diversity analyses, including principal coordinate analysis (PCoA) and PERMANOVA, were performed to evaluate the dissimilarities and similarities between microbial communities across different samples.

Statistical analysis was conducted using R software (version 4.0.3) and various packages, including vegan, ggplot2, and phyloseq. Significant differences in alpha diversity metrics and taxonomic composition among samples were determined using appropriate statistical tests, such as *t*-tests or ANOVA, followed by multiple testing corrections if necessary.

### 2.3. Library Construction and Sequencing

The purified PCR products were quantified using a Qubit^®^ 3.0 fluorometer (Life Invitrogen, Waltham, MA, USA). Amplicons with different barcodes were mixed in equal amounts to create a pooled DNA product. This pooled DNA product was then utilized for the construction of an Illumina paired-end library, following the genomic DNA library preparation procedure provided by Illumina. The constructed amplicon library was subjected to paired-end sequencing (2 × 250) using the Illumina MiSeq platform, sourced from Illumina, Inc., headquartered in San Diego, CA, USA, and this process was carried out by Shanghai Biozeron Co., Ltd., following standard protocols [47]. After the paired-end sequencing process, the raw sequencing data were obtained in the form of FASTQ files. Prior to downstream analysis, quality control of the raw data was performed. This involved trimming and filtering the reads to remove low-quality bases, adapter sequences, and any reads with a high number of ambiguous nucleotides. The processed clean reads were then subjected to bioinformatics analysis. This process included the assembly of paired-end reads, which were merged using programs such as Paired-End reAd mergeR (PEAR). The merged reads were further analyzed for quality control, involving processes such as removing any remaining low-quality bases. For the taxonomic assignment, the clean reads were aligned against a reference database, such as the SILVA database, using an alignment tool like Basic Local Alignment Search Tool (BLAST) or a similar method. Taxonomic classification of the reads was then performed based on their best matches in the reference database. To analyze the diversity and composition of the microbial community, various alpha and beta diversity metrics were calculated. Alpha diversity measures, including the Shannon index and observed species, were used to assess the richness and evenness of the microbial community within each sample. A beta diversity analysis, such as a principal coordinates analysis (PCoA), was applied to examine the dissimilarities or similarities between samples. Statistical analysis was conducted to identify significant differences in microbial composition between samples or experimental groups.

The observed operational taxonomic units (OTUs) were categorized into many taxonomic levels, covering 1 domain, 6 phyla, 35 classes, 123 orders, 344 families, 1279 genera, and 2962 species.

### 2.4. Ethics Policy

Animal rights were strictly observed throughout this study. No research involving human subjects was conducted, and all the samples were collected from woodpecker chests without causing harm to the birds.

Moreover, it is important to mention that the present work was conducted without obtaining or waiving consent from any animal research ethical council. The field sampling procedures employed in this research followed the routine practices of the Bird Ringing Station of Northeast Forestry University, which is authorized and overseen by the Bird Ringing Station of China. The aforementioned processes have received official approval from the relevant governing bodies in order to guarantee the ethical treatment of avian species. It is important to highlight that all the procedures carried out in this study were specifically intended to mitigate stress and prevent any adverse effects on avian biodiversity. Stringent measures were used to guarantee the physical and psychological health of the woodpeckers involved in this study. In order to uphold our dedication to the welfare of animals, we adhered to precise methods aimed at mitigating any potential consequences for the woodpeckers throughout the sample procedure.

## 3. Results

A total of 21 woodpeckers were captured during the entire experimental sampling process, and all the captured woodpeckers’ contour feathers were aseptically sampled. The species composition was as follows: the lesser spotted woodpecker group, comprising eight females and four males; three individuals from the great spotted woodpecker species, including one female and two males; three white-backed woodpeckers, consisting of two females and one male; and three grey-headed green woodpeckers, consisting of one female and two males (Table 1).

### 3.1. α Diversity Indices of Fungi Carried by Each Woodpecker Sample

**Figure 1:**
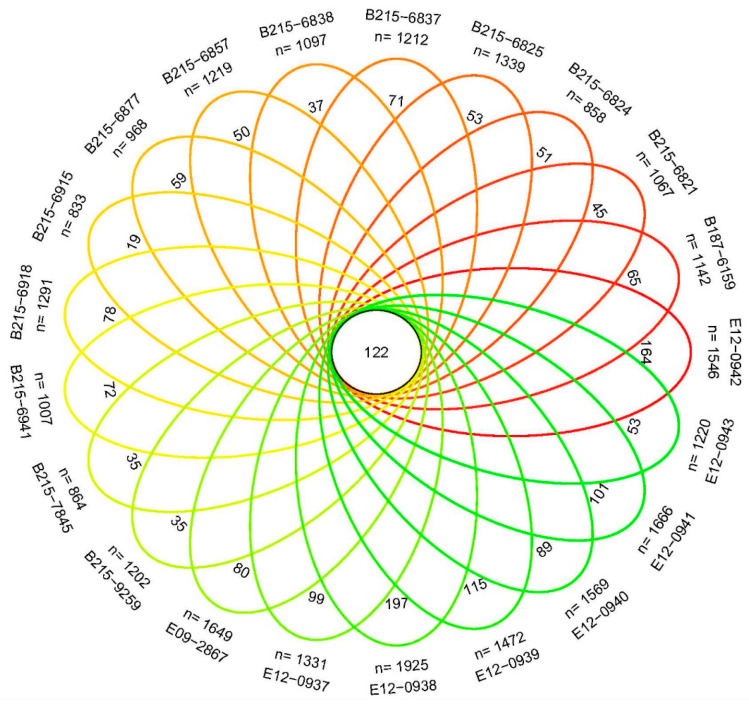
OTU Venn analysis of all samples. The number displayed in the center represents the number of intersecting elements shared by all samples.

The alpha index for each sample in Table 2 can be utilized as a significant indicator of the fungal variety present within the collected samples. The aforementioned techniques have been extensively used for investigation by applying the R software package vegan version 2.5-6 for the purpose of α diversity analysis. In order to ensure the validity of the data, sequence quality control was conducted on the 21 samples of woodpeckers. Afterward, reads of low quality were excluded for the sake of ensuring the optimal analysis of operational taxonomic units (OTUs). The cumulative count of ITS sequences amounted to X bars, with an average sequence length of Y bp. A visual examination of the Venn diagram (Figure 1) revealed the existence of shared OTUs among the samples.

In addition, a comprehensive evaluation of the fungal communities’ richness and diversity among the samples was conducted, utilizing the appropriate α diversity indexes (Table 2). The ACE and Chao indices are essential tools for assessing the richness of fungal communities. Higher values of both indices indicate a greater level of fungal community richness. On the other hand, a lower Simpson index value and a higher Shannon index value are indicators of an elevated level of fungal community diversity within a certain sample. Additionally, Good’s coverage index effectively represents the coverage of a fungal community among samples during the sequencing process. Notably, the results demonstrated that the Good’s coverage index for all samples exceeded 99.9%, affirming the sequencing’s ability to reflect the distinctive characteristics of the fungal community present in the samples. One-way ANOVA was employed to show that there were significant differences between the chao1 and ACE indices among bird species (*p* < 0.05), and Tukey’s HSD post hoc test was used to identify pound-wise differences. Indexes with differences are labeled in Table 2.

Additionally, the ACE and Chao indices revealed that E12-0938 and B215-6824 displayed the greatest and lowest values, respectively, among the samples. This result exhibits the most extreme levels of fungal community richness in each of the sample groups. Additionally, it was found that E12-0938 exhibited the highest degree of fungal diversity, as shown by the Simpson index, while B215-6824 presented the lowest level of fungal diversity, as indicated by the Shannon index. The above-mentioned findings offer significant insights into the prevailing patterns of fungal diversity seen in the collected samples of woodpeckers.

### 3.2. Fungal Diversity Carried by Each Woodpecker

**Figure 2:**
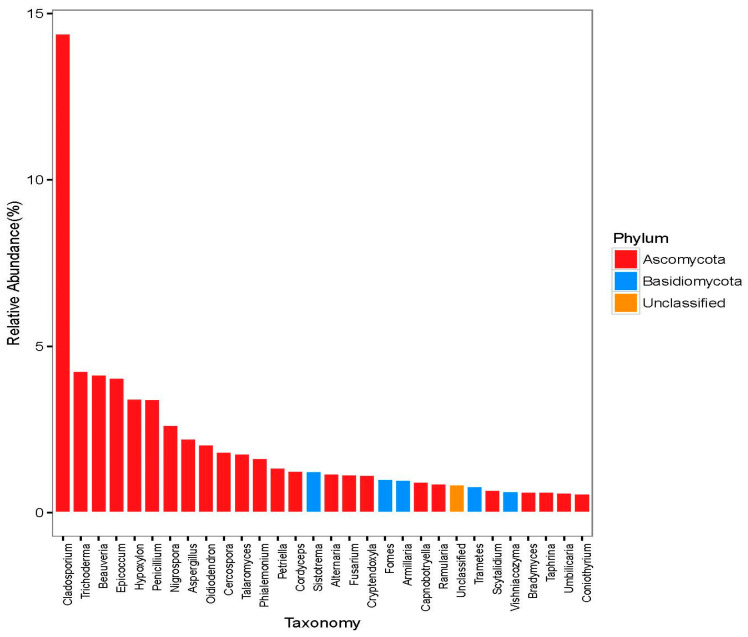
Dominant fungal species carried by the total sample.

Through the application of OTU clustering to ITS gene sequences, a comprehensive total of 5363 distinct species of OTUs was found. These OTUs encompass a wide array of organisms, highlighting the significant diversity present within the dataset. For sequencing, we employed Illumina high-throughput technology, ensuring the extraction of efficient sequence strips. In order to extract significant insights from the data, OTU clustering was conducted with a 97% efficacy criterion for each sample including fungal species. To analyze the fungal species found in the woodpecker samples, the data were evaluated at both the phylum and genus levels. Additional classification analysis revealed that at the phylum level, Ascomycota emerged as the dominant phylum, while Cladosporium stood out as the most prominent genus (Figure 2).

**Figure 3:**
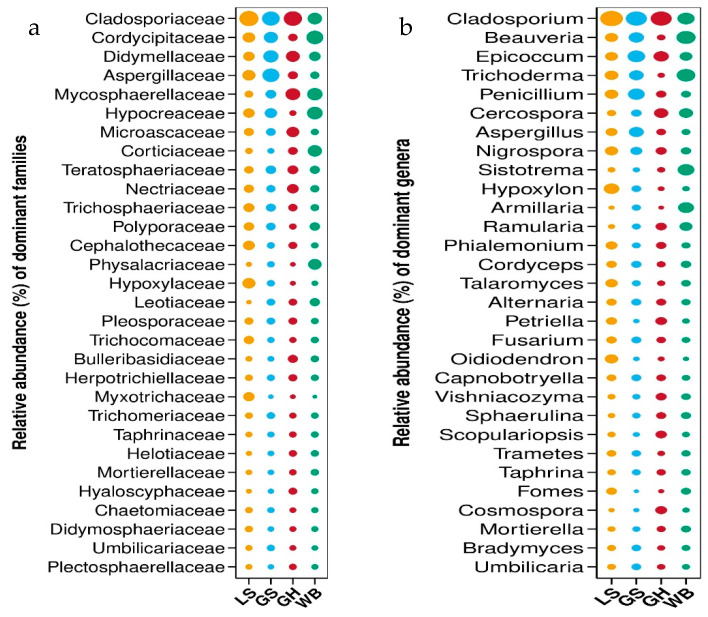
Relative abundance of fungal diversity carried by each woodpecker species, where GH represents the grey-headed woodpecker group, GS corresponds to the great spotted woodpecker group, LS denotes the lesser spotted woodpecker group, and WB signifies the white-backed woodpecker group. (**a**) illustrates the diversity of fungi carried by various bird species at the family level, while (**b**) demonstrates the fungal diversity harbored by these bird species at the genus level.

Significantly, apparent clustering patterns were seen in regard to the fungal species present in the samples obtained from little spotted woodpeckers. To facilitate the effective visualization of these patterns, the R vegan package was utilized to build a heatmap. In the heatmap, a color gradient was applied to depict the distribution of data values, facilitating the detection of clusters that exhibit similarities in terms of species or samples with comparable levels of abundance. This methodology enabled the evaluation of similarities and dissimilarities in community composition across many levels of classification. Figure 3 provides comprehensive graphic representations. 

At the phylum taxonomic level, a notable observation was that Ascomycota demonstrated the greatest species abundance, comprising a substantial 91.96% of the relative species abundance. In a similar vein, it is noteworthy that *Cladosporium* exhibited the greatest proportion of species abundance at the genus level, with a notable percentage of 49.3%. 

After grouping all the samples according to bird species for analysis at the taxonomic level, it was found that the dominant fungal family in the grey-headed woodpecker group was Cladosporiaceae. The top three families in terms of relative abundance were Cladosporiaceae, Mycosphaerellaceae, and Didymellaceae, accounting for 15.043%, 8.438%, and 6.940%, respectively. In the great spotted woodpecker group, the dominant families were Cladosporiaceae and Aspergillaceae. The top four families in terms of relative abundance were Cladosporiaceae, Aspergillaceae, Didymellaceae, and Cordycipitaceae, accounting for 14.147%, 12.585%, 9.212%, and 7.439%, respectively. In the lesser spotted woodpecker group, the dominant family was Cladosporiaceae. The top three families in terms of relative abundance were Cladosporiaceae, Aspergillaceae, and Cordycipitaceae, accounting for 16.659%, 5.846%, and 5.235%, respectively. In the white-backed woodpecker group, the dominant families were Hypocreaceae and Mycosphaerellaceae. The top four families in terms of relative abundance were Hypocreaceae, Mycosphaerellaceae, Cladosporiaceae, and Corticiaceae, accounting for 9.580%, 9.111%, 7.531%, and 7.417%, respectively (Figure 3a).

After grouping all the samples according to bird species for analysis at the genus level, it was found that the dominant fungal genus in the grey-headed woodpecker group was *Cladosporium*. The top three genera in terms of relative abundance were *Cladosporium*, *Epicoccum*, and *Cercospora*, accounting for 13.719%, 5.507%, and 4.428%, respectively. In the great spotted woodpecker group, the dominant genera were *Epicoccum* and *Penicillium*. The top four genera in terms of relative abundance were *Epicoccum*, *Penicillium*, *Beauveria*, and *Aspergillus*, accounting for 8.339%, 7.377%, 5.642%, and 5.206%, respectively. In the lesser spotted woodpecker group, the dominant genus is *Cladosporium*. The top four genera in terms of relative abundance were *Cladosporium*, *Hypoxylon*, *Trichoderma*, and *Penicillium*, accounting for 16.589%, 5.730%, 3.852%, and 3.605%, respectively. In the white-backed woodpecker group, the dominant genera were *Beauveria* and *Trichoderma*. The top four genera in terms of relative abundance were *Beauveria*, *Trichoderma*, *Cladosporium*, and *Sistotrema*, accounting for 10.267%, 9.505%, 7.493%, and 7.004%, respectively (Figure 3b).

### 3.3. A Comparative Analysis of β Diversity among Woodpecker Samples

**Figure 4:**
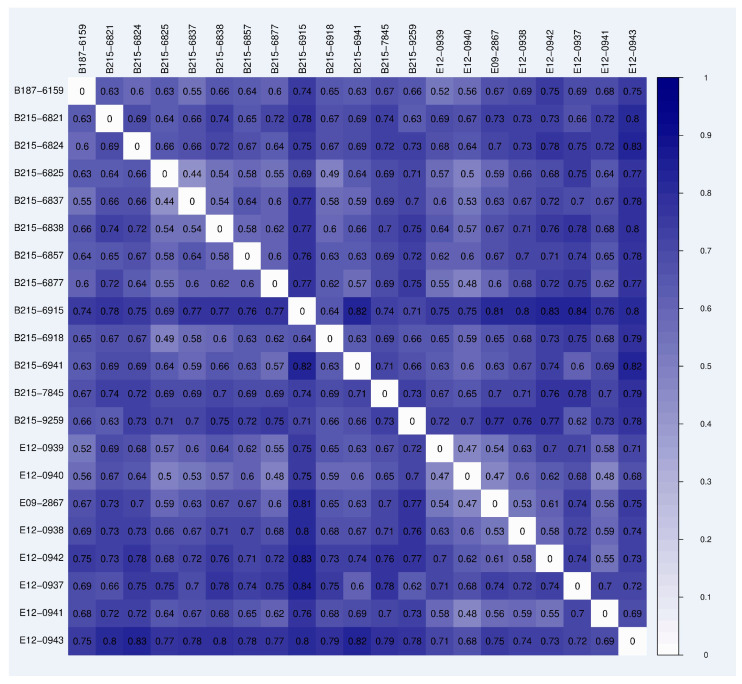
Heatmap analysis of the distance matrix for all the woodpecker samples, with the shade of a color donating distance. The correlations between the samples are expressed using colors, with bluer colors on the color chart indicating greater disparity between correlations.

The Bray–Curtis statistical procedure was employed to compute the distances between the samples, yielding a matrix of distances. Next, the distance matrix was visually represented using a heatmap, facilitating the examination of variations in species distribution among the samples. In the heatmap, distinct colors were utilized to depict individual difference coefficients between two samples, whereby smaller coefficients correspond to lower variances in species diversity (Figure 4).

**Figure 5:**
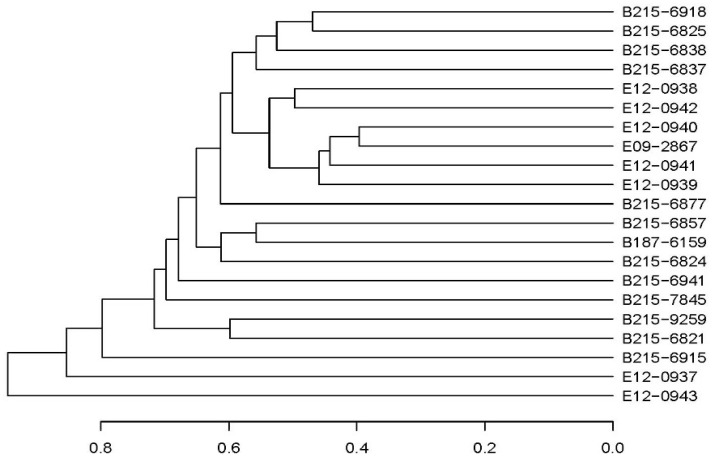
Clustering similar samples based on fungal diversity by using the unweighted pair group method with arithmetic mean (UPGMA) method for clustering.

In order to examine and compare the similarities and dissimilarities among different samples, a hierarchical clustering analysis was performed using the β diversity distance matrix. The tree structure was constructed using the unweighted pair group method with arithmetic mean (UPGMA). The findings indicate that, even following the distance calculation, the fungal groups harbored by different woodpecker species continued to exhibit a tendency to cluster within their respective species. The visualization findings are presented in the form of a tree structure that depicts the links between various samples (Figure 5).

**Figure 6:**
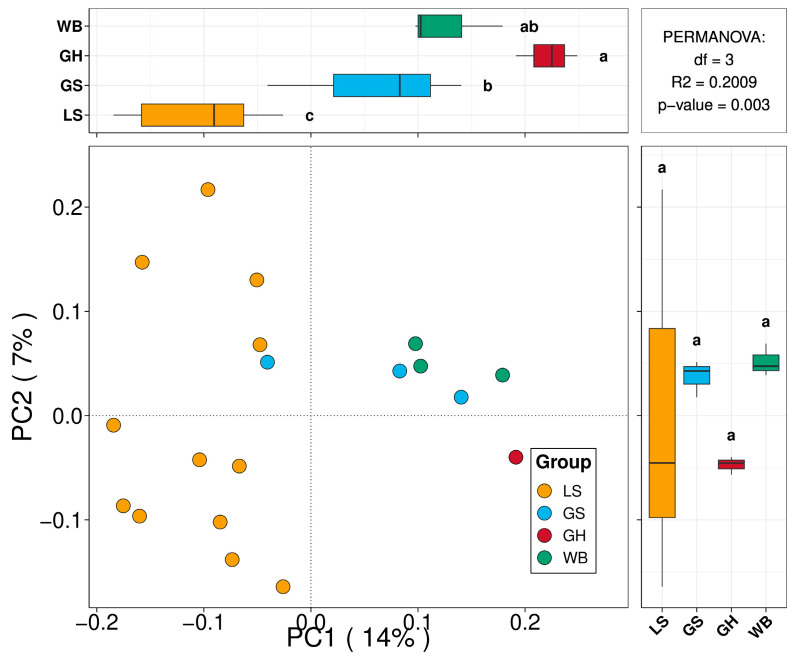
PCoA plot generated based on the Bray–Curtis distances between samples, for which Adonis was used to indicate significant differences in the diversity of fungi carried by woodpeckers among the groups. The different letters indicated represent the various groups to which they belong.

Based on the ITS high-throughput sequencing results, we used the Bray–Curtis distance to measure the similarity or dissimilarity among groups of woodpecker samples, depicting these distances through a principal coordinates analysis (PCoA) plot to reveal underlying relationships or patterns among the samples (Figure 6). A dendrogram was employed to describe and compare the similarities and differences among multiple samples. Initially, the distances between samples were calculated using algorithms that describe the composition and structure of the community based on the beta diversity distance matrix for hierarchical clustering analysis. The unweighted pair group method with arithmetic mean (UPGMA) algorithm was then utilized to construct a dendrogram, generating a tree-like relationship structure for visualization analysis. Statistical significance analysis of the four sample groups was conducted using Adonis. The statistical results indicate significant differences in the diversity of fungi carried by woodpeckers among the groups, with a notable effect (*df*_1_ = 3, *df*_2_ = 17, *F* = 1.44, and *p* = 0.008).

However, it should be noted that there are some limitations of this experiment, including the low sample size obtained from some of the woodpecker species, which may introduce biases to the results. Future studies with larger sample sizes are required to validate our findings.

The PCoA graph provides important insights into the genetic similarities and differences among these 21 woodpecker samples based on the ITS fungal communities within their feathers. The significant clustering pattern of the lesser spotted woodpecker suggests the existence of significant species-specific differences in the ITS fungal community. Meanwhile, the broader variability observed for the grey-headed woodpecker is consistent with the current literature, which suggests that this group has more diverse and variable feeding preferences compared to other species. The PCoA graph offers valuable insights into the genetic affinities and distinctions among woodpecker species, as determined by the ITS fungal communities present in their feathers. The significant clustering pattern observed in the lesser spotted woodpecker indicates the presence of substantial species-specific variations in the ITS fungal community. On the other hand, the wider range of differences seen in the eating habits of the grey-headed woodpecker aligns with existing research that indicates they have a greater variety and range of food preferences compared to other species.

High-throughput sequencing and analysis revealed notable disparities in fungal communities among several woodpecker species, contributing to our knowledge of their genetic variability and ecological functions. Even so, it is crucial to consider the constraints of this experiment, and more research with more extensive sample sizes is necessary to validate the identified patterns.

**Figure 7:**
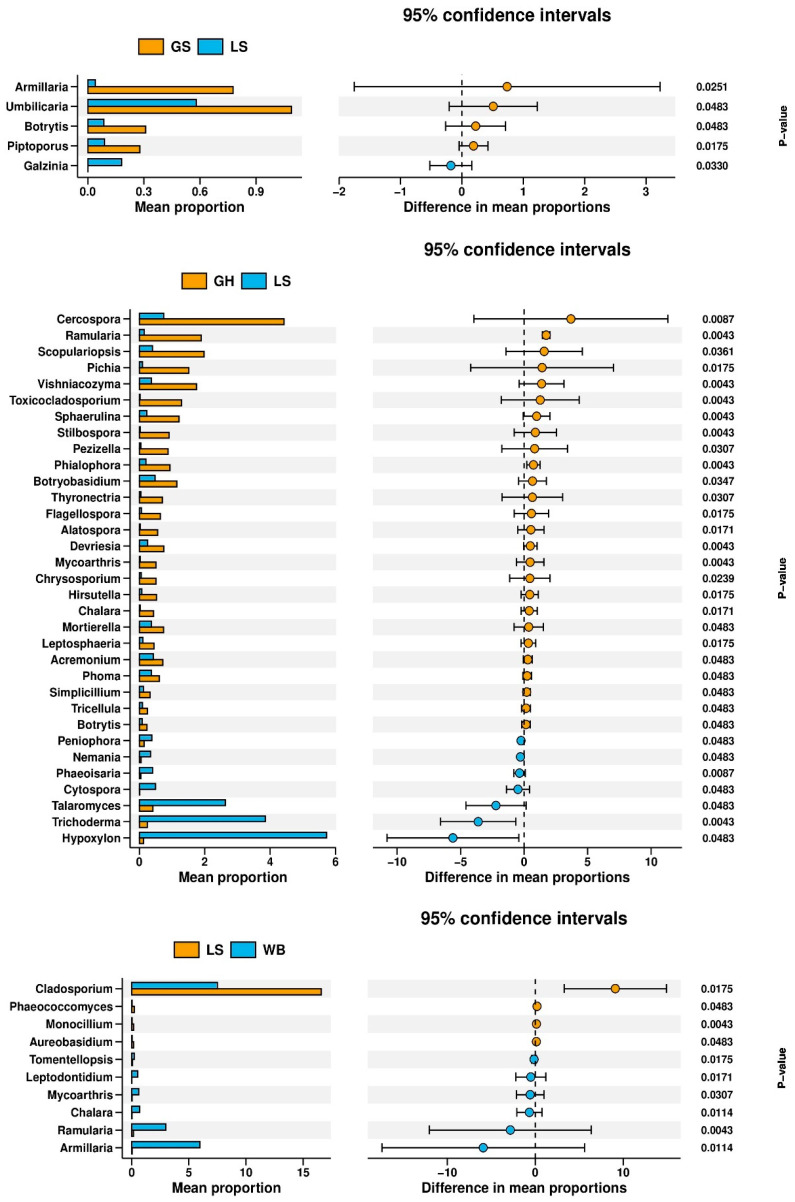
Analyzing differential microbial communities among sample groups at the genus level.

Using the Wilcoxon rank-sum test, significant differences in fungal genera between each pair of sample groups were analyzed at the genus level (Figure 7). At the genus level, no significant differences were found between the grey-headed woodpecker group and the white-backed woodpecker group, the grey-headed woodpecker group and the great spotted woodpecker group, and the great spotted woodpecker group and the white-backed woodpecker group. However, significant differences were observed between the grey-headed woodpecker group and the lesser spotted woodpecker group, specifically in the genera *Acremonium*, *Botrytis*, *Cercospora*, *Chalara*, *Chrysosporium*, *Cytospora*, *Devriesia*, *Hirsutella*, *Hypoxylon*, *Leptosphaeria*, *Mortierella*, *Nemania*, *Peniophora*, *Phaeoisaria*, *Phialophora*, *Phoma*, *Pichia*, *Ramularia*, *Scopulariopsis*, *Simplicillium*, *Sphaerulina*, *Stilbospora*, *Talaromyces*, *Trichoderma*, *Vishniacozyma*, *Alatospora*, *Botryobasidium*, *Flagellospora*, *Mycoarthris*, *Pezizella*, *Thyronectria*, *Toxicocladosporium*, and *Tricellula* (*U* = 6, *p* < 0.05). Significant differences were also found between the great spotted woodpecker group and the lesser spotted woodpecker group in the genera *Armillaria, Botrytis, Piptoporus, Umbilicaria*, and *Galzinia* (*U* = 10, *p* < 0.05). Additionally, significant differences were observed between the lesser spotted woodpecker group and the white-backed woodpecker group in the genera *Armillaria*, *Aureobasidium*, *Chalara*, *Cladosporium*, *Monocillium*, *Phaeococcomyces*, *Ramularia*, *Leptodontidium*, *Tomentellopsis*, and *Mycoarthris* (*U* = 10, *p* < 0.05).

## 4. Discussion

The principal objective of bird ringing stations is to conduct research and examinations of bird habitats and reproductive behaviors while also collecting data pertaining to alterations in ecological variety and the complex migratory patterns exhibited by birds. This was achieved by performing bird population surveys and integrating them with additional qualitative surveys [38,39]. The implementation of such research at bird ringing stations presents various advantages. The personnel conducting field surveys and collecting samples at bird ringing stations have received training, and they have extensive experience in observing and identifying birds. In addition, they also have ample experience in safely removing woodpeckers from mist nets. This approach not only facilitates the gathering of accurate information but also guarantees animal welfare by mitigating any harm to avian species. The use of appropriate protocols guarantees that this process is carried out in a manner that reduces the likelihood of bird fatalities resulting from the incorrect positioning of adhesive bird netting. 

Traditional techniques of fungal identification often rely on the use of isolation cultures and microscopic morphological observation. However, the implementation of these methods and processes requires the expertise of proficient technical experts [48]. In addition, several fungi carried by birds display complex phenotypic traits and provide hurdles in terms of growth on artificial substrates, thereby complicating their identification. The significance of high-throughput sequencing technology in this experiment becomes apparent when considering the limitations and drawbacks of traditional methodologies and microscopic observations. Additionally, the utilization of amplicon technology results in a substantial reduction in the duration of the experimental cycle [49,50], thus offering time-saving benefits. In order to explore the diversity of fungi carried by woodpeckers, regarding sampling methods, in addition to the contour feathers of woodpeckers used in their experiment, NR Johansson et al. chose to use cotton swabs to sample the chest feathers, tail feathers, and feet of woodpeckers [47].

The findings obtained from high-throughput sequencing demonstrated that the phylum Ascomycetes had greater representation in comparison to other phyla, whereas the phylum Basidiomycetes displayed a subdominant presence. The investigation further revealed that the composition of plant endophytic fungi mostly consisted of ascomycetes and basidiomycetes, therefore providing a logical explanation for the experimental results. Ascomycetes also fulfill a vital function in the process of nutrient cycling [51], exhibiting a behavior parallel to that of woodpeckers. Moreover, ascomycetes play a crucial role in maintaining nest stability. This study revealed that the following species numbered among the top 15 most abundant: *Cladosporium*, *Trichoderma*, *Beauveria*, *Epicococcum*, *Hypoxylon*, *Penicillium*, *Nigrospora*, *Aspergillus*, *Oidiodendron*, *and Cercospora*, *Talaromyces*, *Phialemonium*, *Petriella*, *Cordyceps*, and *Sistotrema*. The dominant strain identified in this study was *Cladosporium*, which exhibited notable characteristics, namely, stress and disease resistance [52]. Based on the empirical findings, it is possible to hypothesize that *Cladosporium* also plays a role in enhancing nest stability. In contrast, *Trichoderma* has strong adaptability to its surroundings and has demonstrated its efficacy in exerting biological control against a diverse array of diseases, illustrating the reason behind its existence on bird plumage [53,54].

The fungus *Cladosporium* frequently exhibits stress and disease resistance. Hence, it is logical to hypothesize that *Cladosporium* contributes to the preservation of nest stability. Moreover, the stress and disease resistance characteristics exhibited by *Cladosporium* have the potential to protect woodpeckers’ nests and their offspring against environmental harm and the intrusion of pathogenic microbes [55].

In contrast, *Trichoderma* has a high degree of adaptability to its surroundings and has demonstrated significant biologically mediated control abilities against a range of diseases [56,57]. The capacity to control diseases may play a role in resistance to harmful germs within the nest, hence promoting the maintenance of a healthy and secure nesting environment. Therefore, the identification of *Trichoderma* on bird feathers provides additional evidence for its existence within the nesting habitats of woodpeckers. *Beauveria* and *Cordyceps* are fungal parasites capable of infecting insects [58]. The presence of these fungal organisms may suggest that woodpeckers engage in the consumption of insects that serve as carriers for the spores of these parasitic organisms. *Epicoccum nigrum* is a widely distributed fungus, typically growing on soil, plant residues, and decaying wood. This fungus is renowned for its biocontrol potential, capable of combating various plant pathogens. Therefore, *Epicoccum nigrum* may occupy a higher proportion in the fungal community carried by woodpeckers. Woodpeckers contribute to the mitigation of insect population harm caused by parasites. *Hypoxylon*, *Nigrospora*, *Aspergillus*, *Oidiodendron*, *Phialemonium*, *Petriella*, and *Sistotrema*, among other fungal species, have been identified as potential contributors to the process of wood and organic matter decomposition [59]. Woodpeckers engage in the removal of fungus from wood, a behavior that might result in the transfer of these fungi onto their feathers.

It is important to acknowledge that these interpretations are preliminary speculations derived from preexisting information. Researchers can carry out additional studies and make further observations in order to determine the precise impact and ecological significance of these fungi on woodpecker behavior.

In the context of constructing a UPGMA phylogenetic tree using the Bray–Curtis distance metric, it was noted that the lesser spotted woodpecker exhibited a significant clustering pattern, indicating its distinct grouping. However, the great spotted woodpecker, grey-headed woodpecker, and white-backed woodpecker did not exhibit clear division, suggesting a lack of distinct clustering among these species. This phenomenon can be clarified by several scientific explanations. Firstly, the role of species variety and evolutionary relationships may be of utmost importance. The fungal composition of the lesser spotted woodpecker may exhibit more distinctiveness and specificity, leading to the emergence of a recognizable cluster in the study of operational taxonomic units (OTUs). The observed variations in fungal composition among woodpecker species may be attributed to the ecological behaviors and food choices of the lesser spotted woodpecker. Secondly, it should be noted that the selection of species or operational taxonomic unit (OTU) combinations, together with the choice of distance calculation technique, might have an impact on the outcomes obtained when employing the Bray–Curtis distance metric for UPGMA clustering. Further modification and enhancement of these statistical methodologies might yield more accurate and comprehensive insights into the variations and correlations across various woodpecker species. Lastly, it is important to consider that the size of the sample might also have had an influence on the outcomes. If a larger and more representative sample size of the lesser spotted woodpecker is used, it is possible that the clustering phenomena seen in the operational taxonomic unit study may become more evident. Conversely, inadequate sample sizes or lack of representativeness regarding other woodpecker species might impede their distinction.

In conclusion, the observed variations in outcomes can be traced to a combination of several factors. Further investigation is required to delve into these issues, with the aim of attaining full comprehension of the variations in fungal composition seen among distinct woodpecker species.

Therefore, this investigation considers both the decomposition of wood following fungal infection and the suitability of the community structure within a woodpecker’s nest cavity. However, it is important to acknowledge that this particular form of qualitative survey, which relies on data obtained from environmental monitoring stations, does have certain limitations. When accounting for the home ranges of species of birds, it was observed that only woodpeckers that cross their home range close to the bird ringing station could be captured [60]. The extent to which the survey conducted on these woodpeckers provides an accurate representation of the entire situation, as well as the degree to which the survey findings offered by these woodpeckers are typical, is a subject of debate. The answer to this question is reliant upon the sample size of future investigations. The woodpecker samples that have been taken reflect characteristics associated with woodpecker feeding and nesting. These samples serve as a typical sample of the fungi that are carried by woodpeckers in the surrounding area. Furthermore, they offer valuable insights and potential avenues for further exploration of the interaction between woodpeckers and fungi in future research.

## Figures and Tables

**Table 1:** Coding number, species, and sex of each sampled woodpecker.

Code	Species	Sex
B215-6821	Lesser spotted woodpecker (*Dendrocopos minor*)	Female
B215-6824	Lesser spotted woodpecker (*Dendrocopos minor*)	Female
B215-6825	Lesser spotted woodpecker (*Dendrocopos minor*)	Female
B215-6837	Lesser spotted woodpecker (*Dendrocopos minor*)	Female
B187-6159	Lesser spotted woodpecker (*Dendrocopos minor*)	Female
B215-6838	Lesser spotted woodpecker (*Dendrocopos minor*)	Female
B215-6857	Lesser spotted woodpecker (*Dendrocopos minor*)	Male
B215-6877	Lesser spotted woodpecker (*Dendrocopos minor*)	Male
B215-6918	Lesser spotted woodpecker (*Dendrocopos minor*)	Male
B215-6915	Lesser spotted woodpecker (*Dendrocopos minor*)	Female
B215-9259	Lesser spotted woodpecker (*Dendrocopos minor*)	Female
B215-6941	Lesser spotted woodpecker (*Dendrocopos minor*)	Male
B215-7845	Great spotted woodpecker (*Dendrocopos major*)	Male
E12-0939	Great spotted woodpecker (*Dendrocopos major*)	Female
E12-0940	Great spotted woodpecker (*Dendrocopos major*)	Male
E12-0941	White-backed woodpecker (*Dendrocopos leucotos*)	Male
E12-0943	White-backed woodpecker (*Dendrocopos leucotos*)	Female
E12-0937	White-backed woodpecker (*Dendrocopos leucotos*)	Female
E09-2867	Grey-headed green woodpecker (*Picus canus*)	Male
E12-0938	Grey-headed green woodpecker (*Picus canus*)	Male
E12-0942	Grey-headed green woodpecker (*Picus canus*)	Female

**Table 2:** α diversity index table for all the woodpecker samples. The column names represent the names of each sample, while the row names display the various diversity indices measured for each sample. Columns followed by the same letter do not differ significantly at *p* > 0.05 (Tukey-HSD post hoc test).

Sample	Chao	Richness	Shannon	Simpson	ACE	Evenness	Coverage
B187-6159	1297.485 ^a^	1198	7.0172	0.0284	1285.648 ^a^	0.686191	0.996125
B215-6821	1222.257 ^a^	1067	7.1685	0.0252	1213.468 ^a^	0.712624	0.993377
B215-6824	1023.303 ^a^	939	6.2739	0.0453	1000.803 ^a^	0.635338	0.997798
B215-6825	1678.718 ^a^	1461	6.7221	0.0588	1673.283 ^a^	0.639429	0.993456
B215-6837	1361.550 ^a^	1240	6.7980	0.0503	1377.172 ^a^	0.661542	0.993809
B215-6838	1423.274 ^a^	1177	5.6667	0.0827	1454.921 ^a^	0.555513	0.992303
B215-6857	1433.761 ^a^	1231	6.6452	0.0398	1448.300 ^a^	0.647332	0.991236
B215-6877	1084.000 ^a^	1036	6.6508	0.0429	1080.032 ^a^	0.663969	0.998160
B215-6915	1293.819 ^a^	994	5.1325	0.0910	1317.184 ^a^	0.515467	0.993875
B215-6918	1533.500 ^a^	1424	7.0096	0.0371	1553.191 ^a^	0.669131	0.995846
B215-6941	1186.113 ^a^	1101	6.9438	0.0409	1163.983 ^a^	0.687195	0.997636
B215-7845	1059.146 ^ab^	956	5.0494	0.1758	1029.375 ^ab^	0.509997	0.997586
B215-9259	1424.516 ^a^	1216	6.4727	0.0453	1413.980 ^a^	0.631615	0.991713
E09-2867	2066.640 ^b^	1776	7.3901	0.0312	2097.633 ^b^	0.684628	0.990239
E12-0937	1659.174 ^ab^	1494	6.4693	0.0834	1659.261 ^ab^	0.613499	0.994945
E12-0938	2354.170 ^b^	2151	8.4640	0.0118	2386.452 ^b^	0.764540	0.992978
E12-0939	1800.812 ^ab^	1618	7.1825	0.0395	1810.389 ^ab^	0.673789	0.994098
E12-0940	1891.966 ^ab^	1630	7.2784	0.0359	1906.699 ^ab^	0.682097	0.990140
E12-0941	2318.547 ^ab^	1969	7.4487	0.0242	2343.946 ^ab^	0.680671	0.991523
E12-0942	1770.522 ^b^	1657	7.8700	0.0181	1758.856 ^b^	0.735907	0.995890
E12-0943	1540.224 ^ab^	1244	5.7778	0.0892	1550.103 ^ab^	0.562007	0.989603

## Data Availability

Data are contained within the article.
